# Immediate nipple reconstruction as part of one-stage total breast reconstruction: a vascular design-based narrative review

**DOI:** 10.3389/fonc.2026.1978519

**Published:** 2026-10-07

**Authors:** Ziyi Luo, Yanji Zhang, Hai Li, Xiangkui Wu, Shune Xiao, Chengliang Deng

**Affiliations:** 1 Department of Burns and Plastic Surgery, Affiliated Hospital of Zunyi Medical University, Zunyi, China; 2 Collaborative Innovation Center of Tissue Repair and Regenerative Medicine, Zunyi, China

**Keywords:** autologous breast reconstruction, free perforator flap, immediate nipple reconstruction, local flap, nipple projection, pedicled perforator flap

## Abstract

Autologous breast reconstruction restores breast volume and contour after mastectomy, but reconstruction of the nipple–areola complex remains central to the perception of breast completeness. Traditionally, nipple reconstruction has been delayed until the reconstructed breast mound has stabilized, allowing the final nipple position and projection to be adjusted after soft-tissue settling and scar remodeling. However, in selected patients undergoing autologous reconstruction, simultaneous nipple reconstruction can be integrated into breast shaping, thereby reducing additional procedures, avoiding a second local incision and making use of redundant skin and soft tissue that would otherwise be discarded during flap tailoring. This strategy also allows surgeons to plan breast contour, nipple position, projection and symmetry as a single three-dimensional aesthetic unit. Nevertheless, accurate intraoperative positioning, reliable perfusion and durable long-term projection remain major technical challenges. In this case-based narrative review, we discuss the rationale, potential advantages, patient selection and limitations of immediate nipple reconstruction during autologous breast reconstruction. We further present three representative approaches from our center: a local flap, a pedicled perforator-based flap and a free perforator-based flap. These cases suggest that immediate nipple reconstruction should not be regarded as a single standardized operation, but as a flexible reconstructive strategy that should be tailored to tissue availability, vascular anatomy and aesthetic goals.

## 1 Introduction

Breast cancer remains the most common cancer in women worldwide. In 2022, approximately 2.3 million new female breast cancer cases and 670,000 deaths were estimated globally (1). Surgical treatment is determined by tumor stage, biological subtype, oncological safety and patient preference. For patients with extensive ductal carcinoma *in situ*, invasive breast cancer or contraindications to breast-conserving treatment, mastectomy remains an important therapeutic option. Contemporary mastectomy procedures include skin-sparing mastectomy (SSM), nipple-sparing mastectomy (NSM) and conventional total mastectomy. For patients in whom the tumor does not involve the nipple and subareolar tissue, NSM can be performed. Studies have shown that although NSM can improve appearance to some extent, it carries a risk of necrosis. A French prospective multi-center study of 59 women undergoing prophylactic nipple-sparing mastectomy with immediate breast reconstruction reported complete nipple–areola complex necrosis in 2 patients (3%) and partial or complete necrosis in 9 patients (15%) (2). A study by Jones et al. included 573 patients who underwent SNSM, among whom 23.6% of breasts developed varying degrees of necrosis, and 8 cases developed nipple necrosis with loss of the nipple (3). In the short term, nipple necrosis may lead to nipple loss; in the long term, it may lead to contracture, nipple asymmetry and breast asymmetry, thereby negatively affecting the final appearance of the reconstructed breast.

Post-mastectomy breast reconstruction has become an integral component of breast cancer care, as it contributes not only to restoration of body contour but also to psychological recovery. Within this reconstructive pathway, the nipple-areola complex is a small but visually dominant structure. Its absence often leaves an otherwise successful breast mound looking incomplete. Reconstructing the nipple and areola can therefore help restore body integrity, breast identity and patient satisfaction (4). Techniques for nipple-areola complex reconstruction have evolved substantially. In 1949, Adams described reconstruction using a full-thickness labial graft (5). Subsequent methods have included nipple sharing (6), toe-pulp grafts, auricular cartilage support, dermal-fat flaps, T-flaps, skate flaps, star flaps and C-V flaps. In parallel, medical tattooing has emerged as a minimally invasive method for areolar pigmentation and optical three-dimensional simulation. Becker first reported intradermal tattooing to refine nipple-areola reconstruction in 1986 (7). Medical tattooing has since become an important minimally invasive adjunct for restoring areolar pigmentation and improving the visual completeness of breast reconstruction. More recently, three-dimensional tattooing techniques have been used to create the optical illusion of nipple projection (8, 9). In this review, “immediate nipple reconstruction” refers specifically to surgical creation of the nipple mound during breast reconstruction; areolar reconstruction or pigmentation may be performed simultaneously in selected techniques or as a later staged procedure. Therefore, nipple reconstruction should not be considered synonymous with complete nipple–areola complex reconstruction. Against this background, the timing of nipple reconstruction remains debated. This review examines the theoretical basis, technical feasibility, patient selection and limitations of immediate nipple reconstruction during autologous breast reconstruction, and illustrates three vascular design strategies used in our center: local flap reconstruction, pedicled perforator-based reconstruction and free perforator-based reconstruction. Rather than focusing on a single operative technique, this review proposes a vascular design-based framework for immediate nipple reconstruction, illustrated by three representative cases from our center.

### 1.1 Literature search strategy

A narrative literature search was conducted in PubMed from database inception to September 2026. Search terms included combinations of “nipple reconstruction,” “nipple–areola complex reconstruction,” “immediate nipple reconstruction,” “simultaneous nipple reconstruction,” “single-stage nipple reconstruction,” “one-stage nipple reconstruction,” “breast reconstruction,” “autologous breast reconstruction,” “DIEP flap,” “TRAM flap,” “latissimus dorsi flap,” “local flap,” “perforator flap,” and “free flap.” Additional terms related to vascular design, nipple projection, complications, and patient-reported outcomes were used when appropriate.

Reference lists of relevant original studies and review articles were also manually screened to identify additional publications. English-language publications relevant to the timing, surgical techniques, vascular basis, postoperative projection, complications, aesthetic outcomes, or patient-reported outcomes of nipple reconstruction were considered. As this was a narrative rather than a systematic review, study selection was based on relevance to the clinical and technical scope of the article, and no formal PRISMA-based screening, risk-of-bias assessment, or meta-analysis was performed.

## 2 Traditional delayed nipple reconstruction

The conventional view is that nipple reconstruction represents the final stage of breast reconstruction. Delaying this step allows the reconstructed breast mound to stabilize before a definitive nipple position is selected (10).

Breast reconstruction comprises two principal components: restoration of the breast mound and contour, and reconstruction of the nipple. After mastectomy, many surgeons first prioritize restoration of the breast mound because the final nipple position depends on postoperative breast settling, scar remodeling and the stability of the reconstructed contour (11). If nipple reconstruction is performed too early, subsequent mound migration or scar traction may lead to malposition, bilateral asymmetry or discordance between the nipple and the final breast contour (11, 12). This concern is supported by three-dimensional and longitudinal analyses showing that the skin paddle can change in position and morphology after autologous reconstruction. Iizuka and colleagues reported that the skin paddle may shift laterally and caudally and may increase in apparent size after reconstruction, suggesting that delayed nipple design may need to account for these predictable changes (13). Delayed reconstruction also offers practical advantages. It allows the surgeon to use the contralateral nipple position as a stable reference point, to adjust projection according to the final breast mound and to combine nipple creation with areolar tattooing or secondary revisions.

Although delayed reconstruction is reliable, it is not without drawbacks. Patients must undergo an additional procedure, local anesthesia or further operative planning, and in some cases a second incision on a reconstructed breast surface that has already undergone scar maturation (14). In addition, delayed nipple reconstruction commonly uses local tissue from the anterior surface of the reconstructed breast, which may create local contour changes or distort the breast mound if tissue availability is limited. The delay itself may also influence the patient experience. The absence of nipple can prolong the perception of an incomplete breast and may affect satisfaction with reconstruction, body image and psychosocial adaptation (15). For selected patients, completing the breast mound and nipple reconstruction unit in a single reconstructive stage may reduce psychological burden and avoid the anticipation of another local procedure (16). However, direct comparative evidence regarding psychological benefits and patient-reported outcomes between immediate and delayed nipple reconstruction remains limited.

## 3 One-stage nipple reconstruction

### 3.1 Theoretical advantages

Traditional two-stage nipple reconstruction is usually performed after the breast mound has largely stabilized. Although this approach facilitates later adjustment of nipple position according to changes in breast shape and helps avoid nipple displacement, it also means that patients must undergo an additional operation, a second local incision and potential risks of scar formation and disruption of tissue perfusion (17). By contrast, one-stage reconstruction enables patients to obtain a relatively complete breast appearance earlier after surgery, which may enhance their immediate postoperative sense of bodily integrity and promote psychological adaptation (18). This integrated approach is particularly relevant in autologous reconstruction, in which skin, subcutaneous tissue and perforator-based vascular anatomy can provide multiple tissue layers for nipple construction. In this setting, the nipple can be planned not as an “add-on” after breast mound formation, but as a core component of the complete breast aesthetic unit. Therefore, one-stage nipple reconstruction represents a more integrated reconstructive concept: while reducing additional intervention and potential secondary injury, it emphasizes the synchronous reconstruction of breast form, nipple projection and overall symmetry, providing a theoretical basis for achieving a three-dimensional structure closer to the natural breast (19, 20). However, these advantages remain largely theoretical, and current comparative evidence does not establish superior long-term aesthetic or patient-reported outcomes over delayed reconstruction.

### 3.2 Tissue economy and clinical feasibility

Compared with two-stage reconstruction, one-stage nipple reconstruction can make use of excess tissue generated during flap tailoring while the breast is being shaped. Autologous breast reconstruction often requires trimming of the donor flap to optimize volume, contour and inset. In immediate nipple reconstruction, redundant skin and subcutaneous tissue generated during this process can be redirected into nipple construction. The reconstructed nipple is therefore created within the existing reconstructive field, rather than by reopening the breast mound months later or sacrificing additional local tissue. This is especially applicable to autologous reconstruction, in which the flap itself usually provides sufficient tissue volume and malleability, offering greater flexibility for nipple position, volume and projection (19).

The concept of simultaneous one-stage reconstruction of the breast and nipple can be traced at least to the report by Beegle in 1994 (21), in which immediate TRAM flap breast reconstruction and NAC reconstruction were integrated within the same operative stage, challenging the traditional staged model in which nipple reconstruction is performed only after stabilization of the breast mound. Hudson et al. subsequently reported, in 2001 (22), the feasibility of completing autologous breast and nipple-areola complex reconstruction in a single stage in patients with early breast cancer. Thereafter, Delay et al. (23) explored immediate nipple reconstruction in autologous latissimus dorsi breast reconstruction, Williams et al. (24) in free TRAM flap reconstruction, and Craig et al. (25) in areola-sparing mastectomy combined with DIEP flap reconstruction. These reports indicate that simultaneous nipple reconstruction can be technically feasible across different flap types, mastectomy patterns and vascular contexts. In one-stage autologous breast reconstruction, flap selection should not only restore breast volume and contour, but also provide adequate structural support and reliable vascularity for synchronous nipple reconstruction. Among commonly used autologous flaps, including the DIEP, latissimus dorsi myocutaneous flap, SIEA, PAP and TUG flaps, the DIEP flap offers a favorable combination of tissue volume, pliability, perforator reliability and donor-site morbidity for many patients (26). Its lower abdominal skin and fat can provide substantial soft-tissue thickness for mound shaping and, in selected cases, for simultaneous nipple reconstruction (27, 28). Accordingly, the DIEP flap may represent a relatively ideal platform for combined one-stage autologous breast and nipple reconstruction (29). But the DIEP flap should not be regarded as universally superior; flap choice must be individualized according to body habitus, donor-site availability, vascular anatomy, oncological context and patient preference. The published clinical evidence on immediate or simultaneous nipple reconstruction in the setting of breast cancer–related reconstruction is summarized in **Table 1**.

**Table 1:** Clinical evidence for immediate or simultaneous nipple reconstruction after breast cancer surgery.

Study/design	Patients/indication	Breast reconstruction	Nipple reconstruction	Follow-up	Projection/retention	Complications/revision	Patient-reported outcomes/key limitation
Hudson & Skoll, 2001 (22) Case series	17; selected early breast cancer	Immediate autologous TRAM after skin-sparing mastectomy	Simultaneous local-flap NAC reconstruction (C-V–based)	/	Objective projection NR	Mastectomy-skin-flap necrosis affected cosmesis in some cases;	High satisfaction reported qualitatively; small uncontrolled series
Delay et al., 2006 (23) Retrospective series	30; postmastectomy reconstruction	Autologous latissimus dorsi flap	LD skin paddle folded into a cone + dermal-fat flaps	Mean 16.5 month	7.8 mm at 1 year; 6.8 mm at 2 year	/	70% very good, 23.3% good; small retrospective cohort
Williams et al., 2007 (24) Retrospective comparative	21 immediate; delayed comparator	Free TRAM flap	Immediate vs delayed nipple reconstruction	/	Photographic aesthetic outcomes comparable to delayed group	Complication and revision rates comparable	No validated PROM; incomplete records reduced evaluable sample
Craig et al., 2013 (25) Case series	6; breast cancer, areola-sparing mastectomy	Immediate DIEP flap	Nipple created from DIEP skin paddle	6 month	Position remained stable through 6 mo	No flap/nipple complications; no nipple revisions through 6 month	No validated PROM; very small series and short follow-up
Kim et al., 2013 (30) Case series	5; early breast cancer	Free TRAM or LD + implant	Modified C-V flap after areola-sparing mastectomy	Mean 11 month	Aesthetic result excellent/very good;	2 gutter depressions; 1 small areolar erosion; no skin/areolar necrosis	All patients satisfied; n=5 and short follow-up
Hyza et al., 2015 (31) Retrospective series	17; skin-sparing mastectomy	DIEP or MS-TRAM	Modified Wise pattern with 3 dermal-fat local flaps	Mean 13 month	Aesthetic criteria all ≥7.2/10;	/	Shape 9.7/10; position 9.9/10; 77% very satisfied with sensitivity; selected ptotic/hypertrophic breasts
Hong et al., 2017 (32) Retrospective comparative	61; 37 immediate vs 24 delayed	Two-stage implant reconstruction	At expander-to-implant exchange vs delayed	1 year	Objective projection and symmetry similar at 1 year	/	No validated PROM; immediate group had less radiotherapy exposure
Yoon et al., 2018 (33) Retrospective comparative	51 mastectomy patients	Immediate autologous reconstruction (TRAM/LD)	Classical C-H vs modified C-H flap	1 year	Projection loss ≈50% vs 38%	Complications 26% vs 11%; revisions 17% vs 4%; no complete NAC necrosis	No validated PROM; nonrandomized sequential groups
Hurley et al., 2018 (34) Retrospective cohort	29 patients/34 breasts; mastectomy	Autologous latissimus dorsi ± implant	Immediate local-flap NAC reconstruction	12 month;	Mean projection 6 mm at 1 year in measured subset	No partial/complete nipple necrosis; implant-related complications occurred	BREAST-Q: breast 62, nipple 61, overall 74.3, psychosocial 77.7; 62% response
Cho et al., 2020 (35) Retrospective comparative	17 immediate vs 7 delayed; breast cancer	DIEP flap	Immediate modified C-V vs delayed	4–25 month vs 10–28 month	Photo projection score 3.05 vs 3.03;	No significant difference in complications	No patient PROM; small cohorts and subjective photo scoring
Lee et al., 2021 (19) Prospective SNR + retrospective control	40; 19 simultaneous vs 21 delayed; breast cancer	Autologous LD-based reconstruction	C-V, bipedicled, or two-finger flaps	1 year	Projection ratio vs contralateral: 91% vs 77%	No complications reported in simultaneous group through 1 year	Higher satisfaction across all domains; small nonrandomized study
Stead et al., 2024 (18) Consecutive case series	5; DCIS/LCIS, Wise-pattern SSM	Subpectoral direct-to-implant	Modified Wise-pattern U-shaped local flap	6–12 month	No >25% projection loss at 6 month; slight flattening after radiation in 2	No flap loss/delayed healing; no secondary revision	5/5 positive on 2 nonvalidated satisfaction questions; very small series
Pluta et al., 2025 (36) Single-center cohort	112 patients/117 reconstructions; breast cancer	Implant or tissue expander	Modified rectangular local flap	12 month	Loss of projection 2/117 (1.7%)	Nipple-flap necrosis 4.3%; nipple + skin-edge necrosis 6.8%; implant loss 1.7%; hematoma revision 3.4%	93.1% positive cosmetic opinion among permanent results; no delayed comparator

PS: Nipple survival refers to flap viability without partial or complete necrosis; projection retention refers to the proportion of initial nipple projection maintained during follow-up; projection loss refers to the proportional decrease in nipple projection. These outcomes are not interchangeable.

Overall, the available evidence is derived predominantly from small, single-center observational studies, with considerable heterogeneity in reconstructive techniques, follow-up duration, and outcome assessment. Direct comparative evidence remains limited, particularly regarding nipple malposition after breast settling, long-term projection loss, nipple necrosis, revision burden, operative time, radiotherapy-related changes, potential effects of complications on subsequent adjuvant treatment, and validated patient-reported outcomes. Accordingly, current evidence does not establish the superiority of immediate over delayed nipple reconstruction. Nevertheless, one-stage reconstruction may remain a valuable option in appropriately selected patients, and future studies should focus on standardized patient selection, objective three-dimensional assessment, validated patient-reported outcome measures, and longer-term comparative evaluation.

### 3.3 Technical challenges

The two central challenges of immediate nipple reconstruction are accurate positioning and durable projection. The nipple is the most prominent visual landmark on the breast surface. Successful reconstruction requires not only similar size and shape, but also appropriate position, texture, color and symmetry relative to the contralateral side (37). Even small deviations in nipple position can undermine the aesthetic result, even when the overall breast mound appears acceptable. Traditional two-stage reconstruction usually waits until breast morphology has stabilized before positioning the nipple, precisely to reduce positional error caused by flap descent, scar contraction or post-radiotherapy changes.

Projection loss is the second major challenge. Local flap techniques can achieve satisfactory initial projection, but scar contraction, soft-tissue recoil and tissue thinning can lead to flattening over time. The reported loss of projection can reach 45-75%, which is a major cause of postoperative dissatisfaction and the need for revisional surgery. The main contributing factors include insufficient blood supply to the tissue supporting the nipple, thin tissue and high tension in the flap region. Therefore many surgeons design 25-50% overcorrection at the time of surgery. Over the years, multiple modified flaps have been explored to reduce postoperative loss of nipple projection. Projection loss remains a major challenge after nipple reconstruction. Yoon et al. reported that the mean nipple projection decreased by approximately 50% with the classical C-H flap and 38% with the modified C-H flap at 1 year, with complication rates of 26% and 11% and revision rates of 17% and 4%, respectively (33). Yoo et al. evaluated a different technique, the modified C-V flap with purse-string sutures, and reported higher 12-month projection maintenance than with the conventional C-V flap (60.19% vs. 53.98%), together with a lower revision rate (17.33% vs. 39.02%) (38). The central aim is not only to create a nipple, but to balance position, projection, vascularity and breast proportion over time.

### 3.4 Patient selection

Rational patient selection is essential when nipple reconstruction is performed synchronously during one-stage autologous breast reconstruction. Suitable candidates are those undergoing autologous breast reconstruction with reliable flap perfusion, sufficient soft-tissue volume and safely usable redundant skin or subcutaneous tissue. Patients receiving DIEP, TRAM or other well-vascularized autologous flaps may be considered when tissue thickness, vascular anatomy and aesthetic predictability are favorable. Immediate nipple reconstruction is particularly attractive for patients who wish to reduce the number of surgical stages and obtain a more complete breast appearance early after reconstruction. Conversely, patients with poor vascular conditions, high risk of wound-healing complications, unpredictable postoperative breast morphology or marked risk of radiotherapy-related tissue changes should preferentially undergo delayed nipple reconstruction (28). Additional considerations include previous or planned radiotherapy, smoking, diabetes, vascular disease, obesity, mastectomy-flap perfusion, and the expected degree of postoperative breast settling or ptosis. In unilateral reconstruction, the position and projection of the contralateral nipple should also be considered, whereas bilateral reconstruction allows greater flexibility in symmetric nipple positioning. Importantly, immediate nipple reconstruction is mainly relevant after skin-sparing or total mastectomy when the native nipple is absent or oncologically excised; patients undergoing successful nipple-sparing mastectomy generally do not require nipple reconstruction.

## 4 A vascular design-based framework for immediate nipple reconstruction

Existing evidence for immediate nipple reconstruction remains limited. Prior studies have often focused on individual techniques, such as local flaps or local flaps combined with tattooing, whereas the vascular logic underlying different flap designs has received less attention. To move beyond a purely technique-based classification, we propose a vascular design-based framework that organizes reconstructive options according to the source of vascular support, tissue mobility, and the need for vascular transfer. The following cases from our center illustrate three levels of tissue source and vascular support: local flap reconstruction, pedicled perforator-based reconstruction and free perforator-based reconstruction. The practical value of this framework lies in linking these three reconstructive levels to stepwise intraoperative decision-making, taking into account local tissue availability, perforator position, pedicle length and mobility, the intended nipple position, radiotherapy-related tissue changes, and whether microsurgical vascular anastomosis is required. When postoperative radiotherapy is anticipated or avoidance of additional wound-healing risk before adjuvant treatment is a priority, the timing of nipple reconstruction should be considered cautiously, as evidence regarding the impact of immediate nipple reconstruction on subsequent treatment remains limited.

### 4.1 Local flap nipple reconstruction

Local flaps use skin and subcutaneous tissue adjacent to the intended nipple site to create a projected nipple. They are widely used because they are simple, versatile, do not require an additional donor site and can be customized to the contralateral nipple. Local flaps can be broadly grouped into three patterns. Centrally based flaps, such as C-V and Hammond flaps, elevate a central skin component and wrap surrounding tissue around it to generate projection. Subdermal-pedicle flaps preserve the subdermal vascular plexus and create central projection through rotation or folding; examples include star and diamond flaps. Double subdermal-pedicle designs use two local flaps with preserved subdermal perfusion, which are turned, opposed and sutured to form a nipple. Their proposed advantages include greater tissue volume and preservation of the subdermal vascular plexus, although comparative evidence demonstrating improved flap survival remains limited (39–42).

Our center used a local flap approach for immediate nipple reconstruction during DIEP flap breast reconstruction (**Figure 1**). The method was technically straightforward, did not require a new donor site and added limited operative complexity (40). However, because local flaps depend on surrounding breast tissue, local tissue movement and suture tension may alter the shape of the reconstructed breast. Moreover, follow-up showed that projection can decline over time, consistent with the known limitation of local flap nipple reconstruction (39), which may affect patient satisfaction. Based on these limitations, our center further explored nipple reconstruction techniques based on vascular patterns, with the aim of reducing tissue traction and breast contour changes while achieving more durable maintenance of nipple projection.

**Figure 1:**
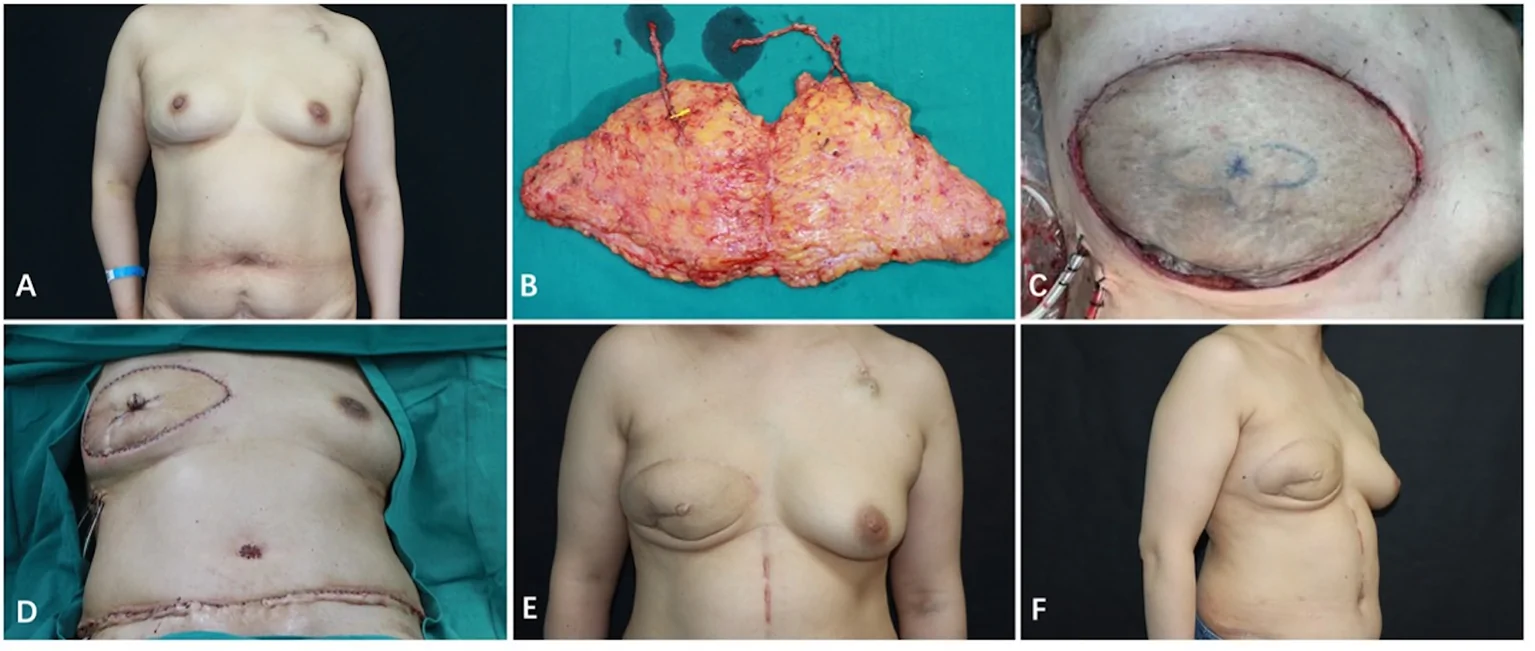
Local flap nipple reconstruction during DIEP flap breast reconstruction. **(A)** Preoperative DIEP flap design and breast markings. **(B)** Intraoperative appearance of bilateral free DIEP flaps. **(C)** Preoperative design for nipple reconstruction using a modified C-V flap. **(D)** Immediate postoperative appearance showing nipple projection and contour. **(E, F)** Nipple appearance at 12 months after surgery.

### 4.2 Pedicled perforator-based nipple reconstruction

Pedicled flap nipple reconstruction refers to the synchronous construction of the nipple during autologous breast reconstruction by using a non-dominant perforator vessel within the flap. The basic principle is to identify and preserve a well-perfused perforator with a clear course in the main breast flap, such as a DIEP or LD flap. The perforator and surrounding soft tissue are then folded to form a nipple column, thereby achieving integrated nipple shaping. Compared with conventional local flap techniques, the blood supply of a pedicled flap nipple is directly supported by the perforator; Compared with conventional local flap techniques, the blood supply of a pedicled flap nipple is directly supported by the perforator. This may theoretically provide more consistent vascular support; however, direct comparative evidence demonstrating lower postoperative necrosis rates remains limited. In addition, unlike the multiple tension sutures required for local flaps, this technique reduces central tissue tension and the risk of distortion, and may therefore offer advantages in maintaining nipple projection and aesthetic outcome. He et al. first reported one-stage nipple reconstruction using a TAP pedicled flap combined with an LD myocutaneous flap in 12 patients with Poland syndrome. Intraoperatively, a crescent-shaped perforator flap was designed on the LD flap and folded centrally to form the nipple. All flaps survived postoperatively, and the mean loss of nipple projection was 29% at 1 year, corresponding to approximately 71% maintenance of the initial projection. Eleven of the 12 patients rated the aesthetic appearance of the reconstructed breast and nipple as very good or good (43). Chen et al. further reported nipple reconstruction using LICAP or AICAP perforator flaps in 21 patients requiring salvage after failure of local flaps. At 6 months postoperatively, nipple projection had decreased by approximately 20%. No postoperative complications were reported, and several BREAST-Q domains improved significantly; the VAS scores for cosmesis and satisfaction with surgery were 9.0 and 9.3, respectively (44).

To our knowledge, clinical reports specifically describing immediate pedicled nipple reconstruction from a secondary perforator within a DIEP flap remain scarce. Based on intraoperative observation and perforator anatomy, our team found that the DIEP flap can contain secondary perforators suitable for axial-design nipple construction. In the case shown in **Figure 2**, a rectangular flap centered on a non-dominant perforator was folded *in situ* to form a nipple, preserving perfusion without adding donor-site morbidity. At 12 months, projection was maintained and the patient was satisfied with both breast and nipple reconstruction.

**Figure 2:**
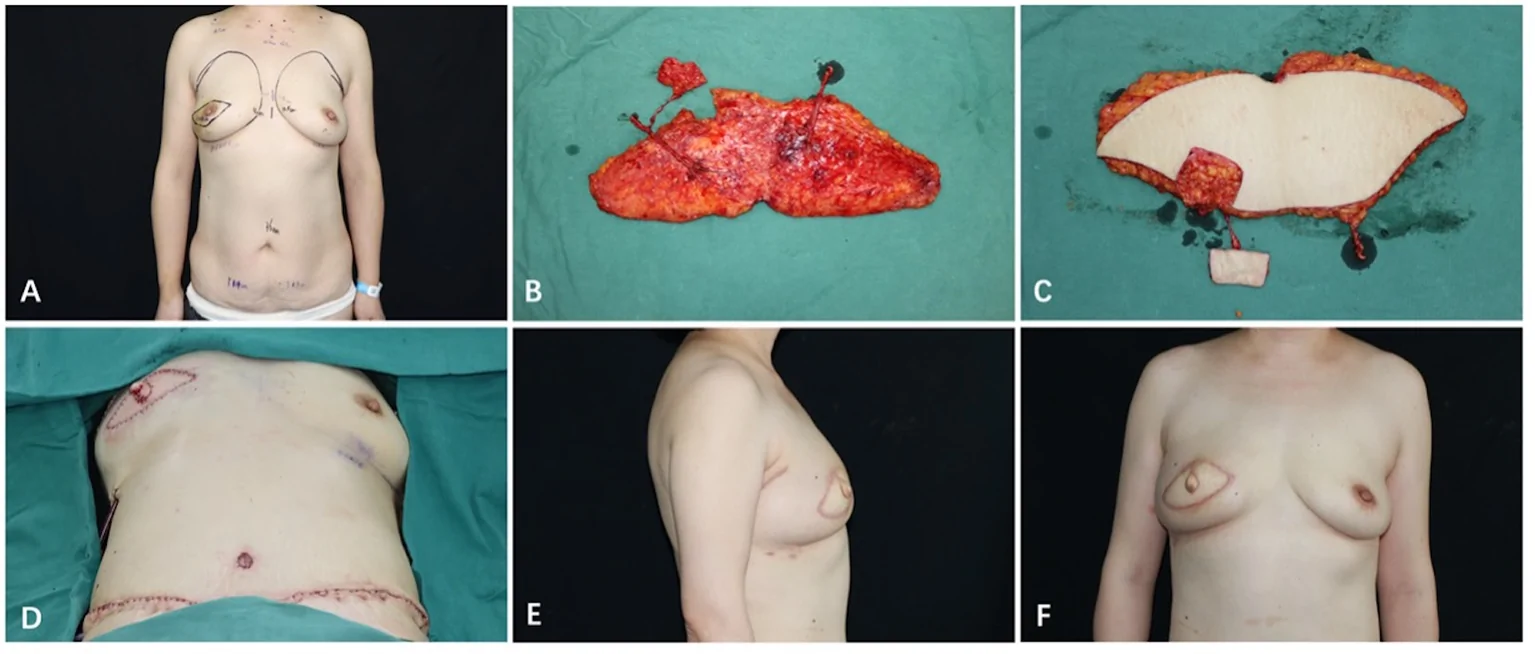
Pedicled perforator-based nipple reconstruction. **(A)** Preoperative DIEP flap design and breast markings. **(B)** Intraoperative appearance of bilateral free DIEP flaps. **(C)** A 6 x 4 cm rectangular flap was designed around a contralateral non-dominant perforator, which served as the vascular source for nipple reconstruction. **(D)** Immediate appearance after nipple reconstruction. **(E, F)** Nipple appearance at 12 months after surgery.

This technique may be considered when the central portion of the flap has sufficient soft-tissue thickness, the perforator anatomy is clear and the surgeon is experienced in perforator-based microsurgical design. However, if the perforator length is insufficient or if the vascular axis deviates from the planned nipple point, intraoperative flexibility in nipple shaping will be markedly limited, potentially leading to nipple malposition and reduced contralateral symmetry (43). In such cases, the planned perforator-based pedicled nipple flap can be converted to a free perforator flap, with microsurgical vascular anastomosis performed at the planned nipple site, thereby overcoming pedicle-length limitations and improving positioning flexibility and reconstructive freedom.

### 4.3 Free perforator-based nipple reconstruction

Free perforator-based nipple reconstruction can be conceptualized in two ways. The first is stacked-flap reconstruction, in which multiple small flaps are used to reconstruct a breast, often combining an abdominal flap with a PAP or TUG flap (45). These additional flaps can contribute local volume and maximal projection and may be used to reconstruct the nipple. Rozen et al. performed bilateral breast reconstruction using stacked DIEP/TUG flaps. On each side, the DIEP flap was used to restore breast volume, whereas the TUG flap was used to increase volume and provide maximal projection; the TUG flap was also used for nipple reconstruction. At 3 months postoperatively, breast volume and contour were satisfactory, and no flap-related complications were observed (46). Similarly, Roggio et al. reported stacked DIEP/TUG breast reconstruction in seven patients, including three unilateral and four bilateral reconstructions. The TUG component contributed additional volume and its skin paddle was used for nipple–areola complex reconstruction. In selected patients, this approach may augment reconstructive volume while providing a vascularized tissue component for nipple–areola complex reconstruction; however, evidence for a specific vascular or long-term projection advantage remains limited (47).

The second approach is a free secondary perforator flap harvested from within the main flap. In this strategy, a small flap based on a secondary perforator is designed within the main DIEP or latissimus dorsi flap. If the perforator is too short or insufficiently mobile for pedicled transfer to the ideal nipple site, the flap can be divided and transferred as a free perforator flap. This avoids an additional donor site while increasing the freedom to position the nipple precisely (**Figures 3**, **4**). The potential technical advantages of this approach include a dedicated vascular supply, adjustable tissue thickness and greater flexibility in nipple positioning. However, these potential benefits have not yet been established in comparative clinical studies. In addition, the small caliber and limited volume of the flap require advanced microsurgical skills, and the procedure may prolong operative time. Preoperative vascular imaging, such as computed tomographic angiography, may be useful in selected cases for identifying suitable perforators and assisting operative planning. This case represents our team’s experience with free perforator flap–based nipple reconstruction. Postoperatively, satisfactory nipple projection was maintained, and the patient reported a high level of satisfaction with the reconstructive outcome.

**Figure 3:**
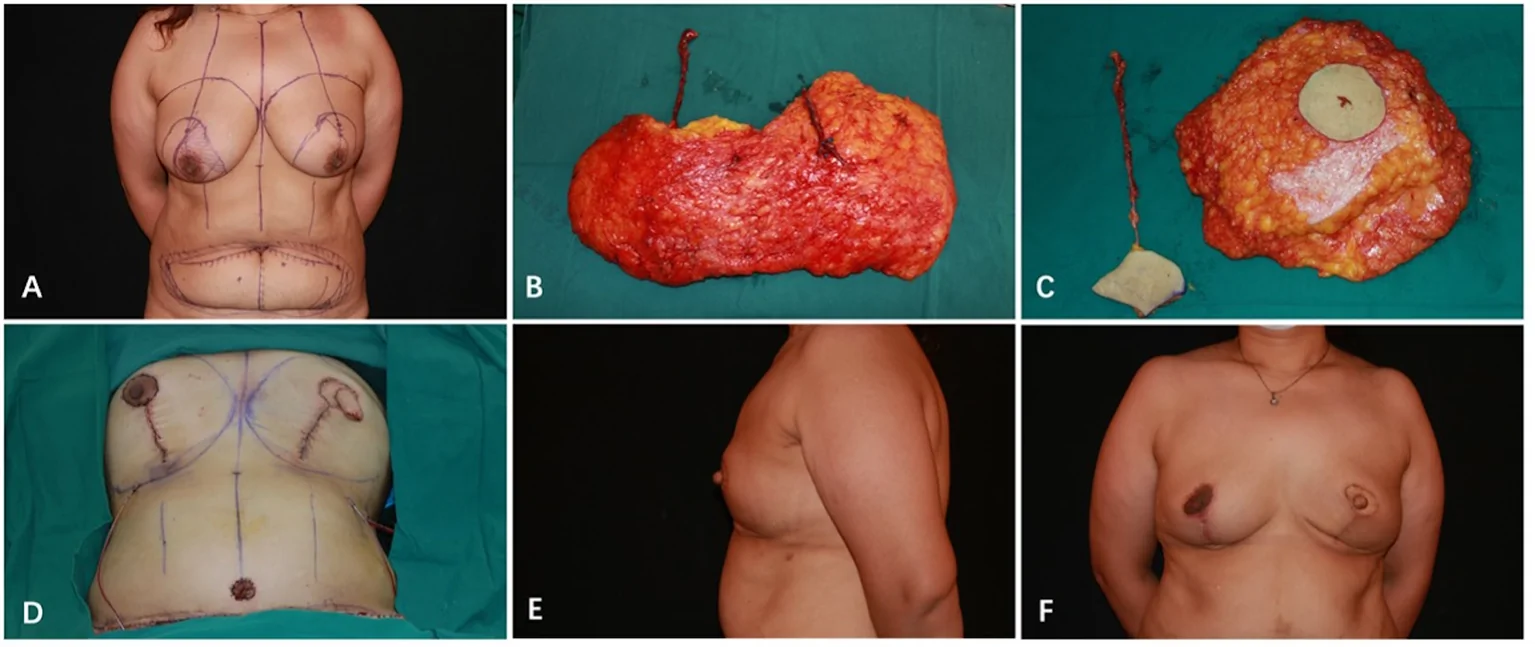
Free perforator-based nipple reconstruction. **(A)** Preoperative DIEP flap design and bilateral breast markings. **(B)** Intraoperative appearance of bilateral free DIEP flaps. **(C)** A 6 x 4 cm rectangular flap centered on a contralateral non-dominant perforator was designed and elevated as a free flap for nipple reconstruction. **(D)** Immediate postoperative appearance, with nipple projection measuring 15.2 mm. **(E, F)** Nipple appearance at 18 months after surgery.

**Figure 4:**
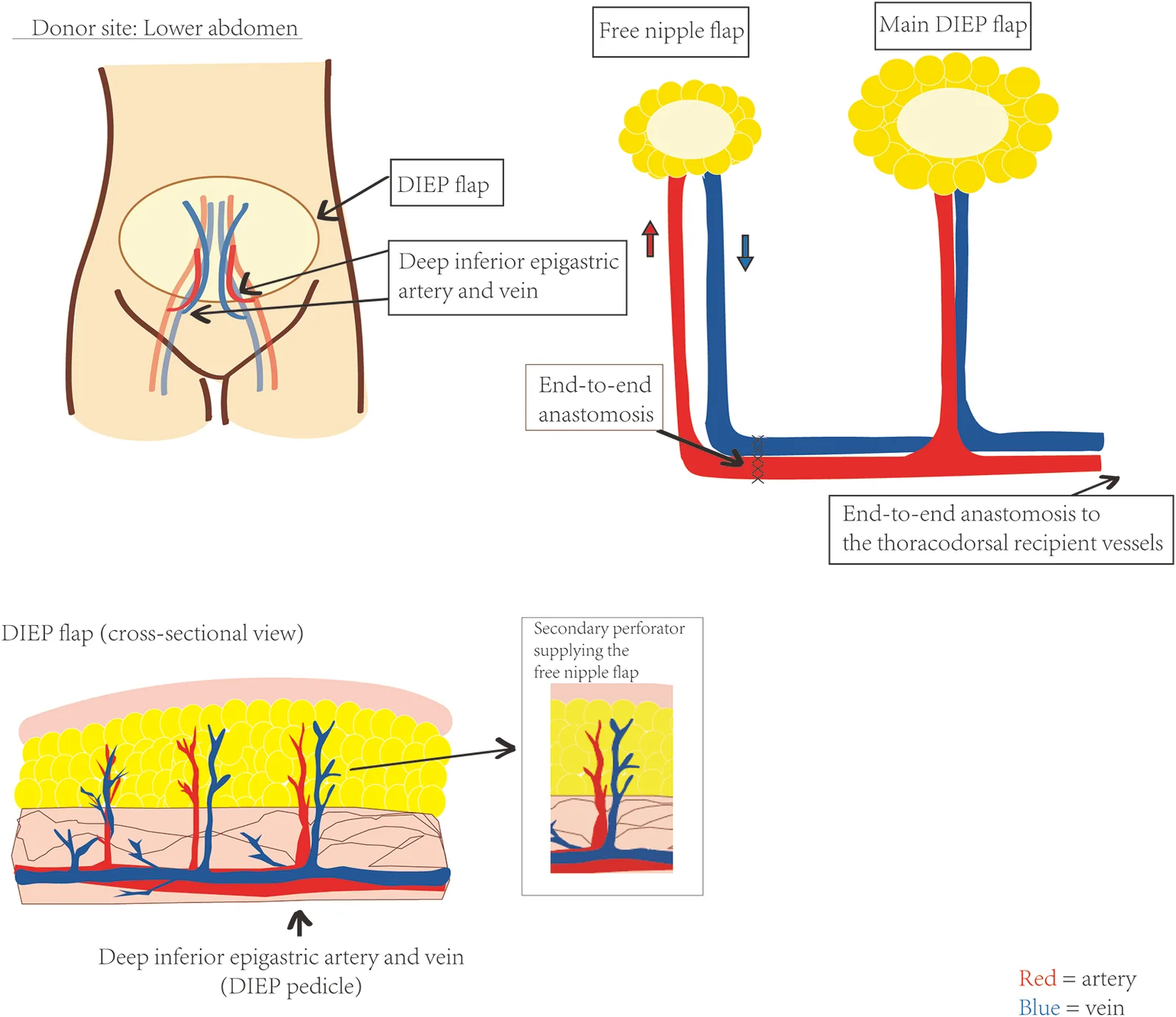
Schematic illustration of free perforator-based nipple reconstruction. A small skin–subcutaneous flap centered on a non-dominant perforator was designed within the lower abdominal DIEP flap as the nipple flap. After elevation of the DIEP flap, end-to-end anastomosis was performed between the nipple flap and the vascular system of the DIEP flap, followed by anastomosis of the opposite end to the recipient vessels. The vascularized nipple flap was then folded and shaped to create nipple projection. Red indicates arteries, and blue indicates veins.

### 4.4 Clinical decision framework

The proposed vascular design-based framework can be translated into a stepwise clinical decision process. First, the feasibility of immediate nipple reconstruction should be assessed according to flap perfusion, tissue quality, anticipated breast-shape stability, and radiotherapy-related considerations. When perfusion is uncertain, substantial postoperative contour change is expected, or radiotherapy is likely to compromise the reconstructed tissue envelope, delayed nipple reconstruction may remain preferable.

When immediate reconstruction is considered appropriate, local tissue availability represents the first decision point. If sufficient well-vascularized skin and subcutaneous tissue are available around the planned nipple site, a local flap provides the simplest option without additional vascular dissection or microsurgical anastomosis.

If local tissue is insufficient and a suitable secondary perforator is available, perforator location, pedicle length and mobility should then be assessed. When the perforator is located close to the intended nipple position and has sufficient pedicle length and mobility, a pedicled perforator-based nipple flap can be used while maintaining its native vascular connection.

If an appropriate perforator is present but its vascular axis or pedicle length does not allow tension-free transfer to the desired nipple position, the flap may instead be converted to a free perforator flap. Microsurgical vascular anastomosis increases positioning flexibility and allows the nipple to be placed independently of the original perforator location, although at the cost of greater technical complexity and operative time.

Accordingly, the choice among local, pedicled perforator-based, and free perforator-based reconstruction reflects a progressive balance between tissue availability, vascular anatomy, positional freedom, and microsurgical complexity. This framework should be regarded as a practical conceptual aid rather than a validated treatment algorithm, and future studies are needed to evaluate its reproducibility and clinical utility.

## 5 Lessons from the cases

Through these cases, we do not aim to compare the superiority or inferiority of different techniques. Rather, we emphasize that one-stage nipple reconstruction can be individualized according to the method of breast reconstruction, available tissue volume and source of blood supply, thereby providing clinical reference for synchronous reconstruction of the breast contour and nipple-areola complex. One-stage nipple reconstruction should not be understood as a single fixed technique; instead, it is closer to an individualized reconstructive strategy based on tissue conditions and vascular patterns. Local flaps, pedicled flaps and free flaps represent different levels of tissue source and vascular basis, and also suggest that nipple construction can be planned synchronously according to the available skin, soft-tissue redundancy and flap malleability during breast reconstruction. In other words, the key to one-stage nipple reconstruction is not simply to perform nipple reconstruction earlier, but to comprehensively assess nipple position, expected projection, tissue redundancy and vascular reliability at the beginning of breast shaping, and to incorporate the nipple-areola complex into the overall design of the complete breast aesthetic unit. Immediate nipple reconstruction should still be applied cautiously. At present, published comparative evidence does not establish a general superiority of immediate over delayed nipple reconstruction. Delayed nipple reconstruction remains more conventional and predictable in many settings, particularly when breast mound settling is expected, perfusion is uncertain or radiotherapy may alter the skin envelope. The evidence base for immediate nipple reconstruction is limited by small case series, heterogeneity of techniques and inconsistent reporting of projection measurements and patient-reported outcomes. This narrative review also has inherent limitations, including a non-systematic literature selection process based primarily on PubMed, potential publication bias toward technically successful cases, heterogeneous methods for measuring nipple projection, limited use of validated patient-reported outcome measures, and the scarcity of robust comparative studies between immediate and delayed reconstruction. Future studies should include larger cohorts, standardized preoperative planning, objective three-dimensional assessment of nipple position and projection, structured patient-reported outcome measures such as BREAST-Q, and long-term follow-up.

## 6 Conclusion

In summary, immediate nipple reconstruction reflects a shift in the concept of breast reconstruction: nipple reconstruction is no longer only a secondary finishing step after breast mound reconstruction, but can be synchronously planned as part of the complete breast aesthetic unit. This shift places unprecedented demands on preoperative planning, tissue design and intraoperative judgment. Careful preoperative planning and intraoperative assessment are important for optimizing flap design in one-stage breast and nipple reconstruction. Although delayed nipple reconstruction remains the more conventional and reliable choice at present, previous clinical reports suggest that, with strict case selection, synchronous nipple reconstruction during autologous breast reconstruction is feasible. Its potential advantages include reducing additional operations, more effectively using redundant tissue generated during breast shaping, and integrating breast contour and nipple projection within the same three-dimensional framework. The cases from our center further suggest that immediate nipple reconstruction is not a fixed procedure, but an individualized strategy that can be adjusted according to different tissue sources and vascular patterns, including local flaps, pedicled flaps and free flaps. Nevertheless, this technique should still be applied with caution. Accurate judgment of nipple position, reliability of blood supply and long-term maintenance of projection remain key challenges. Future studies with larger sample sizes, standardized aesthetic assessments and longer follow-up are needed to clarify its indications, morphological stability and practical value compared with delayed nipple reconstruction.

## References

[1] Kim J Harper A McCormack V Sung H Houssami N Morgan E . Global patterns and trends in breast cancer incidence and mortality across 185 countries. Nat Med. (2025) 31:1154–62. doi: 10.1038/s41591-025-03502-3 39994475

[2] Houvenaeghel G Cohen M Dammacco MA D’Halluin F Regis C Gutowski M . Prophylactic nipple-sparing mastectomy with immediate breast reconstruction: results of a French prospective trial. Br J Surg. (2021) 108:296–301. doi: 10.1093/bjs/znaa082 33793719

[3] Jones VM Nelson JA Sevilimedu V Moo TA . Impact of mastectomy flap necrosis on patient-reported quality-of-life measures after nipple-sparing mastectomy: a preliminary analysis. Ann Surg Oncol. (2024) 31:6795–803. doi: 10.1245/s10434-024-15681-3 38990221 PMC11684468

[4] Mantovani GP Blessent CGF Federici GP Taccioli F Pinelli M . Yin-Yang NAC sharing: full-thickness split graft from a single NAC for bilateral immediate reconstruction after skin-reducing mastectomy. JPRAS Open. (2026) 48:6–11. doi: 10.1016/j.jpra.2025.10.029 41362495 PMC12682058

[5] Adams WM . Labial transplant for correction of loss of the nipple. Plast Reconstr Surg. (1949) 4:295–8. doi: 10.1097/00006534-194905000-00009 18131359

[6] Millard DR . Nipple and areola reconstruction by split-skin graft from the normal side. Plast Reconstr Surg. (1972) 50:350–3. doi: 10.1097/00006534-197210000-00006 4561575

[7] Becker H . The use of intradermal tattoo to enhance the final result of nipple-areola reconstruction. Plast Reconstr Surg. (1986) 77:673. doi: 10.1097/00006534-198604000-00032 3952225

[8] Hammond JB Teven CM Bernard RW Lucas HD Casey WJ Siebeneck ET . 3D nipple-areolar tattoo: it's technique, outcomes, and utilization. Aesthetic Plast Surg. (2021) 45:453–8. doi: 10.1007/s00266-020-01967-w 32968821

[9] Allam O Foster C Knoedler L Knoedler S Oh SJ Pomahac B . Future of autologous breast reconstruction: a review of novel technological innovations. Plast Aesthet Res. (2024) 11:19. doi: 10.20517/2347-9264.2024.21

[10] Losken A Duggal CS Desai KA McCullough MC Gruszynski MA Carlson GW . Time to completion of nipple reconstruction: what factors are involved? Ann Plast Surg. (2013) 70:530–2. doi: 10.1097/SAP.0b013e318281ac61 23542858

[11] Fuentes PM Lascar E Sharma S Chowdhury S Lee R Singh D . A review of nipple-areola complex reconstruction and tattooing techniques. Gland Surg. (2026) 15:25. doi: 10.21037/gs-2025-aw-522 41668920 PMC12884090

[12] Tsuruta Y Yano T Kuramoto Y Suesada N Fuse Y Karakawa R . Breast shape evaluation after free flap breast reconstruction after more than 10 years follow-up using 3-dimensional imaging device. Ann Plast Surg. (2022) 88:555–9. doi: 10.1097/SAP.0000000000003015 34611089

[13] Iizuka C Matsumine H Sakurai H . Changes in skin paddle morphology after autologous breast reconstruction. Plast Reconstr Surg Glob Open. (2024) 12:e6175. doi: 10.1097/GOX.0000000000006175 39301309 PMC11412706

[14] Tierney BP Hodde JP Changkuon DI . Biologic collagen cylinder with skate flap technique for nipple reconstruction. Plast Surg Int. (2014) 2014:194087. doi: 10.1155/2014/194087 25114802 PMC4120924

[15] Momoh AO Colakoglu S de Blacam C Yueh JH Lin SJ Tobias AM . The impact of nipple reconstruction on patient satisfaction in breast reconstruction. Ann Plast Surg. (2012) 69:389–93. doi: 10.1097/SAP.0b013e318246e572 22868326

[16] Doyle B Shaari E Hamed H Kothari A . Outcomes after skin-reducing mastectomy and immediate hybrid breast reconstruction using combination of acellular dermal matrix and de-epithelialized dermal flap in large and/or ptotic breasts. Ann Breast Surg. (2022) 6:12. doi: 10.21037/abs-21-10

[17] Yang S Edalatpour A Israel J Gast K . Recent trends in nipple reconstruction - a case series and social media analysis of patient preferences. J Plast Reconstr Aesthet Surg. (2025) 108:1–7. doi: 10.1016/j.bjps.2025.06.042 40669114

[18] Stead TS Francalancia S Laspro M Tanney K Larson B . Immediate nipple reconstruction in skin-sparing mastectomy with a modified wise-pattern design. Plast Reconstr Surg Glob Open. (2024) 12:e5979. doi: 10.1097/GOX.0000000000005979 39027893 PMC11257668

[19] Lee JH Ryu JY Lee JH Lee J Park HY Yang JD . Simultaneous nipple reconstruction in autologous breast reconstruction. Gland Surg. (2021) 10:2966–77. doi: 10.21037/gs-21-338 34804884 PMC8575712

[20] Mughal M Berner JE Ho-Asjoe M See M Roblin P Rose V . One-stop autologous breast reconstruction: a safe and effective cost-saving pathway. J Plast Reconstr Aesthet Surg. (2024) 92:276–81. doi: 10.1016/j.bjps.2023.05.048 38582053

[21] Beegle PH . Immediate single-stage TRAM and nipple-areola reconstruction. Clin Plast Surg. (1994) 21:321–6. doi: 10.1016/S0094-1298(20)30755-0 8187425

[22] Hudson DA Skoll PJ . Single-stage, autologous breast restoration. Plast Reconstr Surg. (2001) 108:1163–71. doi: 10.1097/00006534-200110000-00009 11604613

[23] Delay E Mojallal A Vasseur C Delaporte T . Immediate nipple reconstruction during immediate autologous latissimus breast reconstruction. Plast Reconstr Surg. (2006) 118:1303–12. doi: 10.1097/01.prs.0000244013.11974.ec 17051099

[24] Williams EH Rosenberg LZ Kolm P de la Torre JI Fix RJ . Immediate nipple reconstruction on a free TRAM flap breast reconstruction. Plast Reconstr Surg. (2007) 120:1115–24. doi: 10.1097/01.prs.0000279142.46729.94 17898584

[25] Craig ES Walker ME Salomon J Fusi S . Immediate nipple reconstruction utilizing the DIEP flap in areola-sparing mastectomy. Microsurgery. (2013) 33:125–9. doi: 10.1002/micr.22020 22903381

[26] Farajzadeh MM Salibian AA . Optimizing perfusion and volume in autologous breast reconstruction: dual-plane, conjoined and stacked flaps. Plast Aesthet Res. (2024) 11:32. doi: 10.20517/2347-9264.2024.24

[27] Maruccia M Magistri S Lucarelli N Gurrado A Testini M Giudice G . From breast cancer to DIEP mommy makeover: an onco-aesthetic reconstructive approach. Plast Reconstr Surg. (2026). doi: 10.1097/PRS.0000000000013192 42153733

[28] Tušak M Porčnik A Kneževič I Markovič Božič J Tušak M Lapoša A . Breast reconstruction after cancer: historical development, modern techniques, and psychological impact. Healthcare (Basel). (2026) 14:1140. doi: 10.3390/healthcare14091140 42121583 PMC13164038

[29] Garoosi K Kelson K Winocour J Mathes D Kaoutzanis C . Modern approaches to alternative flap-based breast reconstruction: stacked deep inferior epigastric perforator flaps. Plast Aesthet Res. (2024) 11:42. doi: 10.20517/2347-9264.2024.57

[30] Kim HR Lim JS Kim SM Jung SN Yoo G Rha EY . One-stage nipple and breast reconstruction following areola-sparing mastectomy. Arch Plast Surg. (2013) 40:553–8. doi: 10.5999/aps.2013.40.5.553 24086809 PMC3785589

[31] Hyza P Streit L Vesely J Stafova D Sin P . New technique of immediate nipple reconstruction during immediate autologous DIEP or MS-TRAM breast reconstruction. Ann Plast Surg. (2015) 74:645–51. doi: 10.1097/SAP.0000000000000006 25136923

[32] Hong KY Kim YE Minn KW Jin US . Immediate nipple reconstruction during implant-based breast reconstruction. Aesthetic Plast Surg. (2017) 41:793–9. doi: 10.1007/s00266-017-0804-4 28204930

[33] Yoon JS Chang JW Ahn HC Chung MS . Modified C-H flap for simultaneous nipple reconstruction during autologous breast reconstruction: surgical tips for safety and cosmesis. Med (Baltimore). (2018) 97:e12460. doi: 10.1097/MD.0000000000012460 30235735 PMC6160055

[34] Hurley CM McArdle A Joyce KM O'Broin E . Skin-sparing mastectomy with immediate nipple reconstruction during autologous latissimus dorsi breast reconstruction: a review of patient satisfaction. Arch Plast Surg. (2018) 45:534–41. doi: 10.5999/aps.2017.01725 30466233 PMC6258984

[35] Cho HJ Kwon HJ Moon SH Jun YJ Rhie JW Oh DY . One-stage nipple and breast reconstruction using a deep inferior epigastric perforator flap after a skin-sparing mastectomy. Arch Plast Surg. (2020) 47:26–32. doi: 10.5999/aps.2019.00598 31964120 PMC6976742

[36] Pluta P Piekarski JH Zadrożny M . A novel technique for immediate nipple reconstruction with a rectangular flap in implant-based breast reconstruction. Arch Med Sci. (2025) 21:153–9. doi: 10.5114/aoms/141229 40190323 PMC11969540

[37] Zucca-Matthes G Urban C Vallejo A . Anatomy of the nipple and breast ducts. Gland Surg. (2016) 5:32–6. doi: 10.3978/j.issn.2227-684X.2015.05.10 26855906 PMC4716863

[38] Yoo H Park S Chang H . Nipple reconstruction using modified C-V flap with purse-string sutures for maintenance of long-term nipple projection. J Plast Reconstr Aesthet Surg. (2023) 84:62–70. doi: 10.1016/j.bjps.2023.04.061 37327734

[39] Dolmans GHCG van de Kar AL van Rappard JHA Hoogbergen MM . Nipple reconstruction: the “Hammond” flap. Plast Reconstr Surg. (2008) 121:353–4. doi: 10.1097/01.prs.0000300297.72188.e4 18176266

[40] Yang JD Ryu JY Ryu DW Kwon OH Bae SG Lee JW . Our experiences in nipple reconstruction using the Hammond flap. Arch Plast Surg. (2014) 41:550–5. doi: 10.5999/aps.2014.41.5.550 25276648 PMC4179360

[41] Solis Bermudez U Monroy Velazquez MG Lara Sampayo KL Estrada Tijerina SF Trujillo Rodriguez JE Ruiz Lopez MA . Modified C-V flap for nipple reconstruction. Cureus. (2025) 17:e80256. doi: 10.7759/cureus.80256 40070889 PMC11893373

[42] Gougoutas AJ Said HK Um G Chapin A Mathes DW . Nipple-areola complex reconstruction. Plast Reconstr Surg. (2018) 141:404e–16e. doi: 10.1097/PRS.0000000000004166 29481412

[43] He J Xu H Wang T Qiao Y Zhang Y Dong J . Immediate nipple reconstruction with thoracodorsal artery perforator flap in breast reconstruction by latissimus dorsi myocutaneous flap in patients with Poland's syndrome. Microsurgery. (2016) 36:49–53. doi: 10.1002/micr.22395 25752505

[44] Chen S Yang Q Zheng S Chen C Gao L Liu J . Evaluation of the efficacy and safety of pedicled perforator flap technique for salvage nipple reconstruction in breast cancer patients: a pilot study. Surg Innov. (2024) 31:263–73. doi: 10.1177/15533506241242906 38571331

[45] Steele TN Teotia SS Haddock NT . Multi-flap microsurgical autologous breast reconstruction. J Clin Med. (2024) 13:5324. doi: 10.3390/jcm13175324 39274536 PMC11396626

[46] Rozen WM Patel NG Ramakrishnan VV . Increasing options in autologous microsurgical breast reconstruction: four free flaps for “stacked” bilateral breast reconstruction. Gland Surg. (2016) 5:255–60. doi: 10.3978/j.issn.2227-684X.2015.06.06 27047791 PMC4791351

[47] Roggio T Pantelides NM Morgan M Ramakrishnan V . Stacked TUG and DIEP flaps to reconstruct a single breast: expanding the scope of autologous breast reconstruction. J Plast Reconstr Aesthet Surg. (2022) 75:2974–81. doi: 10.1016/j.bjps.2022.06.039 35906160

